# The Impact of Education and Physical Therapy on Oral Behaviour in Patients with Temporomandibular Disorder: A Preliminary Study

**DOI:** 10.1155/2021/6666680

**Published:** 2021-01-25

**Authors:** Lili Xu, Bin Cai, Shenji Lu, Shuai Fan, Kerong Dai

**Affiliations:** ^1^Department of Rehabilitation Medicine, Ninth People's Hospital, Shanghai Jiao Tong University School of Medicine, Shanghai, China; ^2^Department of Rehabilitation Medicine, Fengcheng Hospital of Fengxian District of Shanghai, Shanghai, China; ^3^Department of Orthopaedics Medicine, Ninth People's Hospital, Shanghai Jiao Tong University School of Medicine, Shanghai, China

## Abstract

Patient education is important in the treatment of temporomandibular disorder (TMD), but little is known about its effect on oral behaviors. We aimed to determine the dominant oral behaviours in patients with TMD and assess the impact of education on such behaviours. Between July 2018 and April 2019, 54 patients diagnosed with TMD according to DC/TMD were recruited. They received physical therapy and were provided education on TMD and offered a list of recommendations for improving their oral behaviours. The patient education process usually lasted for 10–20 min. Of these patients, 48 were reexamined at the outpatient clinic, 3–9 months posttreatment. We recorded the Oral Behaviour Checklist (OBC) score, maximum painless mouth opening (mm), visual analogue scale (VAS) score for pain, and Jaw Functional Limitation Scale (JFLS) score pre- and posttreatment. Wilcoxon signed rank test and paired sample *t*-test were used for statistical analysis. Results showed that the most dominant oral behaviours included “putting pressure on the jaw” (59.3%); “chewing food on one side” (46.3%); “pressing, touching, or holding teeth together at times other than eating” (33.3%); and “eating between meals” (33.3%). Posttreatment, the patients reported a decrease in “chewing gum” (*P* = 0.002), “leaning with the hand on the jaw” (*P* = 0.013), “chewing food on one side” (*P* ≤ 0.001), and “eating between meals” (*P* = 0.007), but this change was not significant in subgroups with a follow-up interval of 9 months. We also observed a significant improvement in the maximum painless mouth opening (*P* ≤ 0.001), JFLS score (*P* ≤ 0.001), and VAS score (*P* ≤ 0.001) for pain, posttreatment. In conclusion, patient education can facilitate management of oral behaviours and should be targeted towards specific oral behaviours.

## 1. Introduction

Temporomandibular disorder (TMD) is a common orofacial pain condition. It refers to a group of disorders characterised by pain in the temporomandibular joint (TMJ), periarticular area, or mastication muscles; noise from the TMJ during mandibular movements; and deviations or restrictions in the mandibular range of motion. The aetiology of TMD is multifactorial, including, but not limited to, factors, such as trauma, occlusal pathology, and oral behaviours (OBs) [1].

According to research evidences, certain OBs are associated with TMD, such as muscle tightening and tensing, diurnal teeth-grinding, and sustained talking [2–4]. OBs may affect the masticatory structures, resulting in peripheral nociceptive disorders, as well as pain and functional disorders [3]. In addition, they may also result in microtrauma of the temporomandibular disc and joint.

Previous studies on OBs in patients with TMD mainly focused on individual OBs (e.g., lip and nail biting, teeth clenching, and playing mouth instruments). Moreover, they lacked detailed descriptions of the frequencies of OBs, thus making comparisons across studies difficult. Use of the Oral Behaviour Checklist (OBC) ensures consistency in OB assessment [5]. The checklist semiquantitatively scores 21 items that represent OBs according to their frequency of occurrence. It has been recommended by the 2014 version of the Diagnostic Criteria for TMD (DC/TMD) for the screening of OBs [6].

Patient education is an important aspect of a comprehensive treatment plan for those with TMD [7–10]. In addition, different education methods may have varied impacts on the treatment outcomes. According to Kang et al. [11], incorporating an educational video or leaflet to the traditional procedure of patient education (oral explanation) increased patient compliance and satisfaction with a self-exercise programmer. Furthermore, counselling and self-management-based therapies can be considered conservative, low-cost, and beneficial treatment alternatives for TMD [12]. Some studies on patient education aimed at improving OBs have reported the impact on reduction of TMD-related pain [13–15]. However, only a few studies have explored the ability of patient education and counselling to change OBs in patients with TMD. According to the current understanding of the disease, physical therapy can improve the symptoms of TMD patients, such as limited mouth opening and pain, which was used as a routine treatment in this study [16–19]. In this study, we aimed to determine the dominant type of OBs in patients with TMD. In addition, we also aimed to explore changes of the dominant OBs after patient education in patients with TMD.

## 2. Materials and Methods

Our study was approved by the Ethics Committee of our hospital, Shanghai Jiao Tong University School of Medicine, Shanghai, China (approval date: 17-10-2016; approval no.: 2016-114-T63). The study procedures conformed to the standards set forth in the Declaration of Helsinki. We received informed consent from all patients after providing them an explanation of the study procedures.

### 2.1. Study Setting and Participants

We recruited 54 patients with TMD who had received physical therapy at our hospital between July 2018 and April 2019. The inclusion criteria were as follows: (1) a diagnosis of TMD according to the DC/TMD [6] and (2) completion of the OBC. Patients were excluded if they had the following characteristics: (1) a history of head or face trauma in the past 10 years; (2) systemic diseases, such as psoriatic arthritis, rheumatoid arthritis, gout, or other diseases affecting the masticatory system; or (3) inflammatory, oncologic, or viral diseases of the face, jaw, spine, or skeletal muscles. The patients were assessed at baseline and 6 months after the last treatment session.

### 2.2. Oral Behaviour Checklist

The patients completed the OBC during their first treatment session at the Rehabilitation Department of our hospital. The OBC is a self-reported scale for identifying and quantifying the frequency of jaw overuse behaviours. We provided explanations for each item in the OBC before the checklist was filled in. Any questions or concerns of the patients were answered and clarified appropriately.

The OBC consists of 21 items representing OBs, each with five possible answers. A scoring system of 0–4 points was used based on the degree of behaviours or habits as follows: 0, none of the time; 1, a little of the time; 2, some of the time; 3, most of the time; and 4, all of the time. The first two OBs occurring at night were scored as follows: 0, none of the time; 1, <1 night/month; 2, 1 to 3 nights/month; 3, 1 to 3 nights/week; and 4, 4 to 7 nights/week. For example, a patient could achieve a score from 0 to 4 for “clenching teeth” as follows: 0, none of the time; 1, a little of the time; 2, some of the time; 3, most of the time; and 4, all of the time. A score of 3 or 4 would indicate the presence of this OB.

### 2.3. Clinical Examination

A doctor familiar with the DC/TMD form and with more than 5 years of experience in treating patients with TMD examined all study participants. They were asked specific questions about pain at rest, during mouth opening and chewing, and examined for the opening and closing of the mouth, vertical range of motion, and location of pain during mouth opening. We recorded the maximum painless mouth opening (mm) and the general visual analogue scale (VAS) score for pain before and after the treatment. In addition, we used the Chinese version of the Jaw Functional Limitation Scale (JFLS) [20] to evaluate jaw function pre- and posttreatment.

### 2.4. Physical Therapy

All patients received physical therapy, including ultrasound therapy [18] (1.0 W/cm^2^, 5 min, 3 MHz), low-frequency pulse laser therapy [19] (880 nm,10 min), manual therapy [16], posture training [17], and muscle relaxation exercises [18]. The frequency of the physical therapy was 1–3 times a week, and it was conducted for 40 min per session. The patients were subjected to 3–10 consecutive treatments.

### 2.5. Patient Education

All patients were educated on TMD. The doctor and the therapist in charge of the patient's treatment provided an explanation for the causes, treatment options, and prognosis of TMD during the first session. The OBC was used to identify the possible risk factors (e.g., teeth clenching and chewing gum) for TMD. In addition, the patients were provided with a customised list of recommendations that covered the following objectives: (1) learning the normal resting position of the TMJ to rest the masticatory muscles; (2) observing and reducing parafunctional habits; (3) avoiding excessive mandibular movements, such as wide opening of the mouth; (4) maintaining a soft diet by cutting food into smaller pieces, and chewing carefully; (5) performing simultaneous bilateral mastication; and (6) improving posture.

The patient education process usually lasted for 10–20 min. According to the evaluation results of OBC, the corresponding content of patient education will be emphasized. For example, if the patient is used to clenching the teeth, the therapist will explain more to the patient how to keep the jaw relaxed. They were taught skills to maintain their TMJ in the normal resting position and improve their posture. Any questions or concerns about the recommendations were discussed with the doctor or therapist until all concepts were completely understood.

### 2.6. Statistical Methods

This study's outcome measure was a participant's follow-up OBC score. We performed statistical analyses using SPSS Statistics for Windows, Version 23 (IBM Corp., Armonk, NY). Moreover, we conducted the Wilcoxon signed rank test to compare the frequency of each OB pre- and posttreatment. Descriptive statistics are presented as median values. The paired sample *t*-test was used to compare the total OBC score, mean maximum painless mouth opening, JFLS score, and VAS score before and after the treatment. A *P* value < 0.05 was considered statistically significant.

## 3. Results

Table 1 summarises the prevalence of each OB. Forty-eight of the 54 included patients returned for the second evaluation at the outpatient clinic, 3–9 months after the last treatment session. We lost six patients to follow-up, with two not answering the phone and four refusing to return because of the long travelling distance. Due to the large gap in the time interval between the second assessment, the patients were divided into three subgroups of 3 months, 6 months, and 9 months for analysis.

The patients comprised 8 men and 46 women. The mean age was 31.94 ± 12.27 years (16–72 years). 12 patients were diagnosed as having masticatory muscle pain, and 42 patients were diagnosed as having disc displacement. While more than half of the patients with TMD (59.3%) had the habit of “sleeping in a position that put pressure on the jaw,” 46.3% patients had the habit of “chewing food on one side” (Table 1). In addition, 33.3% patients had the habits of “pressing, touching, or holding teeth together at times other than eating” and “eating between meals.” Furthermore, 31.5% reported the habit of “yawning.” The sum of all OBC scores was calculated and divided as follows [21]: before treatment, 0–16 (29.2%), 17–24 (40.8%), and ≥25 (33.3%), and after treatment, 0–16 (27.1%), 17–24 (35.4%), and ≥25 (37.5%).

Repeated-measure analysis of the total OBC scores did not reveal a significant difference before and after treatment (*P* = 0.96, Table 2). However, analysis of each OBC item showed different results (Table 3). There was no significant difference in OBs among the three subgroups at baseline (*P* > 0.05).Whilst there was an increase in “clenching or grinding teeth when asleep” and “placing tongue between the teeth,” we observed a decrease in “chewing gum,” “leaning with the hand on the jaw,” “chewing food on one side,” “eating between meals,” and “singing” in subgroups with a second assessment at 3 or 6 months (*P* < 0.05 for all; Table 3). Except for “chewing gum,” there was no significant difference in oral behaviours between the second assessment and the baseline assessment in the subgroup with the interval between two assessments being 9 months (*P* > 0.05; Table 3). Moreover, there was a significant improvement in the mean maximum painless mouth opening, JFLS score, and pain VAS score at the follow-up than in those obtained at baseline (*P* ≤ 0.001 for all; Table 2).

## 4. Discussion

The present study found that certain OBs, such as “putting pressure on the jaw”; “chewing food on one side”; and “pressing, touching, or holding teeth together at times other than eating” were dominant in patients with TMD. Patient education and physical therapy brought about substantial improvements in the maximum painless mouth opening, jaw function score, and pain score. In addition, the patients reported decreases in some OBs, namely, “chewing gum,” “leaning with the hand on the jaw,” “chewing food on one side,” “eating between meals,” and “singing.”

Certain OBs, such as holding, tightening, or tensing muscles, grinding teeth together during waking hours, and sustained talking, can reportedly increase the risk of TMD [22]. However, previous studies on OBs in TMD have examined only a few of them (e.g., chewing gum, bruxism, or thumb sucking) [23, 24]. Ohrbach [25] developed the OBC, which assesses the characteristics of various OBs, thus facilitating their complete assessment in one study. In addition, the OBC is scored on a scale of 0–4 according to the frequency of the different OBs. This in turn enables performing semiquantitative analyses of different OBs. OBs were only described by patient responses of “yes” or “no” in previous studies. However, the OBC is a semiquantitative scoring method. Therefore, it accurately reflects the characteristics of a patient's habits, thus facilitating data processing and analysis.

TMD is a condition caused by many factors, with OBs being possible risks. However, few studies have evaluated the behavioural changes concomitant with TMD. We observed a significant improvement in the maximum mouth opening, jaw function, and pain level after treatment in this study. Simultaneously, there were decreases in “chewing gum,” “leaning with the hand on the jaw,” “chewing food on one side,” “eating between meals,” and “singing.” These changes may have been related to the effects of patient education. However, the patients also reported an increase in “clenching or grinding teeth when asleep” and “placing tongue between the teeth.” This in turn can be attributed to the increased attention paid to the aforementioned OBs by the patients. However, this effect was not significant in subgroups with a follow-up interval of 9 months. 33.3% of patients with TMD had a total OBC score of ≥25 before treatment. A case-control study [21] involving 3,448 subjects found that the corrected odds ratio for an OBC score of >25 was 16.8, thus suggesting OBs to be significant risk factors for TMD. There was no significant difference in the total OBC score before and after treatment in our study. This can be attributed to concomitant increases in some OBs and decreases in others.

Patient education and counselling have been shown to improve pain intensity and oral health-related quality of life in patients with TMD [26]. A systematic review comparing the impact of counselling and other self-management therapies, with interocclusal appliances, for the management of myofascial TMD, reported equivalent effects on spontaneous pain, muscle tenderness on palpation, and maximum opening (with and without pain) [12]. Moreover, the combined application of counselling with posture training and physical therapy programmes provided better results than when used alone [27]. Patient education and counselling consisted of providing patients with details on the aetiological factors, treatment options, and prognosis of TMD, as well as recommendations for behavioural changes in our study. Patient education was found to change the OBs of patients with TMD, but the effect subsided after 9 months. Psychological factors, including psychological distress, pain catastrophizing [28], fear-avoidance beliefs [29], depressed or anxious mood [30], and passive coping [31], are related to increased pain perception and an augmented level of disability in patients with chronic and painful TMD. Patient education and counselling may improve the understanding of the disease, thereby eliminating its associated fear. This in turn may contribute to disease recovery. Therefore, it is essential to redefine the recommendations according to the self-management strategies for different types of TMD and to address the associated psychosocial impairments [32].

Despite the efficacy of patient education and behavioural management along with physical therapy, the patients may undoubtedly benefit from occlusal appliances and occlusal therapy, in conjunction with physical therapy and OB changes. Therefore, TMD treatment should focus on thorough occlusal, TMJ, muscular, and behavioural strategies to comprehensively manage this complex disorder for obtaining the best outcomes. In other words, TMD management is dependent on detailed and extensive intra- and extraoral evaluations across multiple disciplines. Thus, the findings of this study elucidated the significance of patient education in the management of TMD.

However, our failure to include a healthy control group was the main limitation of this study. This prevented a comparative evaluation of the differences between the patients and the healthy subjects. Moreover, our study lacked a control group that did not receive education and physical therapy, thus preventing careful interpretation of the results. Future research should focus on the relationship between OBs and patient education and the changes of OBs at different time points.

## 5. Conclusions

Patients with TMD exhibited OBs, such as “putting pressure on the jaw”; “chewing food on one side”; “pressing, touching, or holding teeth together at times other than eating”; “eating between meals”; and “yawning.” Patient education and physical therapy applied together may change the oral behaviours of patients with TMD, but this effect basically disappeared 9 months after intervention. Therefore, in the future, more research should be carried out to observe the effect of patient education on oral behaviour at different intervals.

## Figures and Tables

**Table 1:** Prevalence of oral behaviours in patients with temporomandibular disorder, before and after physical therapy and patient education.

Oral behaviour	Before (*N* = 54)		After (*N* = 48)	
		Prevalence (%)		Prevalence (%)
(1) Clenching or grinding teeth when asleep	11	20.4	14	29.2
(2) Sleeping in a position that puts pressure on the jaw	32	59.3	25	52.1
(3) Grinding teeth	0	0	1	2.1
(4) Clenching teeth	3	5.6	1	2.1
(5) Pressing, touching, or holding teeth together at times other than eating	18	33.3	10	20.8
(6) Holding, tightening, or tensing muscles without clenching or bringing teeth together	5	9.3	5	10.4
(7) Holding or jutting jaw forward or to the side	7	13	3	6.2
(8) Pressing tongue forcibly against the teeth	3	5.6	2	4.2
(9) Placing tongue between the teeth	5	9.3	12	25
(10) Biting, chewing, or playing with the tongue, cheeks, or lips	3	5.6	0	0
(11) Holding the jaw in a rigid or tense position, such as to brace or protect the jaw	6	11.1	2	4.2
(12) Holding between the teeth or biting objects	2	3.7	1	2.1
(13) Using chewing gum	2	3.7	0	0
(14) Playing musical instruments that involve use of the mouth or jaw	0	0	0	0
(15) Leaning with the hand on the jaw	10	28.5	0	0
(16) Chewing food on one side	25	46.3	7	14.6
(17) Eating between meals	18	33.3	7	14.6
(18) Sustained talking	14	25.9	6	12.5
(19) Singing	2	3.7	2	4.2
(20) Yawning	17	31.5	6	12.5
(21) Holding the telephone between the head and shoulders	2	3.7	0	0

**Table 2:** Comparison of the maximum painless mouth opening, VAS score for pain, and JFLS score, before and after physical therapy and education for patients with TMD.

	Before treatment	At 3–9 months after treatment	*P* value^∗^
Maximum painless mouth opening (mm)	25.6 ± 7.5	37.2 ± 4.8	**<0.001**
VAS score for pain	2.94 ± 2.27	1.05 ± 1.74	**≤0.001**
JFLS score	52.42 ± 31.52	32.23 ± 28.29	**≤0.001**

Note: data are presented as the mean ± standard deviation. ^∗^The values in bold indicate significant differences between values before treatment and those after treatment (*P* < 0.05). VAS: visual analogue scale; JFLS: Jaw Functional Limitation Scale; TMD: temporomandibular disorder.

**Table 3:** Comparison of oral behaviours before and after physical therapy and education for patients with TMD.

Oral behaviour	Before (*N* = 54)		After (*N* = 48)		3 months (*N* = 9)		6 months (*N* = 29)		9 months (*N* = 10)	
	Med	*P*	Med	*P*	Med	*P*	Med	*P*	Med	*P*
Item 1	0		2	**0.002**	2	**0.026**	2.	**0.017**	2	1.000
Item 2	3		3	0.906	2	0.524	3	0.502	3	1.000
Item 3	0		0	0.102	0	1.000	0	0.157	0	0.317
Item 4	1		1	0.693	1	0.206	1	0.542	1	0.257
Item 5	2		1	0.089	1	0.054	1	0.356	1	0.942
Item 6	1		1	0.197	0	0.157	1	**0.032**	1	1.000
Item 7	0		0	0.388	0	0.518	0	0.142	1	0.595
Item 8	0		0	0.297	0	1.000	0	0.655	0	0.059
Item 9	0		1	**0.009**	1	0.119	1	**0.021**	0	0.713
Item 10	0		0	0.491	0	0.655	1	0.435	0	0.577
Item 11	0		1	0.369	0	1.000	1	0.427	1	0.713
Item 12	0		0	0.491	0	0.317	0	0.109	0	0.317
Item 13	0		0	**0.002**	0	0.157	0	0.070	0	**0.038**
Item 14	0		0	1.000	0	1.000	0	1.000	0	1.000
Item 15	1		1	**0.013**	1	**0.039**	1	**0.049**	1	1.000
Item 16	2		2	**≤0.001**	2	**0.016**	1	**0.005**	2	0.851
Item 17	2		2	**0.007**	2	0.161	2	**0.073**	1	0.096
Item 18	1		2	0.137	2	0.435	2	0.194	2	0.803
Item 19	1		1	**0.008**	2	0.234	1	**0.005**	1	0.915
Item 20	2		2	0.056	2	0.861	2	0.109	2	0.096
Item 21	0		0	0.377	0	0.317	0	0.454	0	0.317

Note: data are presented as the median values. The values in bold indicate significant differences between values before treatment and those after treatment (*P* < 0.05). Med: median; TMD: temporomandibular disorder.

## Data Availability

The SAV data used to support the findings of this study are restricted by the Ethics Committee of the Ninth People's Hospital, Shanghai Jiao Tong University School of Medicine in order to protect patient privacy. Data are available from Lili Xu (heblll@163.com) for researchers who meet the criteria for access to confidential data.
